# Percutaneous Mechanical Thrombectomy for Acute Limb Ischemia With Aorto-iliac Occlusion

**DOI:** 10.3389/fsurg.2022.831922

**Published:** 2022-04-26

**Authors:** Xinrui Yang, Xiangxiang Li, Minyi Yin, Ruihua Wang, Kaichuang Ye, Xinwu Lu, Weimin Li, Yong Cheng, Jinbao Qin

**Affiliations:** ^1^Department of Vascular Surgery, School of Medicine, Shanghai Ninth People's Hospital, Shanghai JiaoTong University, Shanghai, China; ^2^Vascular Center of Shanghai JiaoTong University, Shanghai, China; ^3^Department of Vascular Surgery, People's Hospital of Fuyang, Fuyang, China

**Keywords:** aortic bifurcation embolism, acute limb ischemia (ALI), Rotarex catheter, endovascular treatment (EVT), percutaneous mechanical thrombectomy (PMT)

## Abstract

**Background:**

To evaluate the outcomes of percutaneous mechanical thrombectomy (PMT) with Rotarex catheter in patients with acute lower limb ischemia (ALI) caused by aorto-iliac occlusion.

**Materials and Methods:**

Data of patients with ALI caused by aorto-iliac occlusion in our institutions from January 2010 and April 2020 were reviewed. The primary end point was limb salvage rate. The secondary end points included technical success rate, survival rate, complications after the operation and during the follow-up.

**Results:**

A total of 85 patients with ALI was diagnosed with aorto-iliac occlusion. Thirty-eight patients were treated by PMT with Rotarex catheter and enrolled in present study. Twenty-four were male (63.2%), and 14 were female (36.8%). The mean age was 66 years (range 28–83). All 38 patients were treated with PMT, with additional catheter directed thrombolysis (2/38, 5.3%), balloon angioplasty (8/38, 21.1%) and stent deployment (7/38, 18.4%). The mean procedure time was 123 ± 31 min. Seven patients (18.4%) underwent continuous renal replacement therapy. Two patients received major amputations (above the knee) and 2 patients died for renal insufficiency and heart failure during the hospital stay. Thirty-day survival rate was 94.7% and limb salvage was 94.4%. The mean follow-up time was 14.0 months (8–22 months). There was no major amputation and target artery occlusion occurred during the follow-up period.

**Conclusion:**

PMT with Rotarex catheter could be new option for acute aorto-iliac occlusion, leading to safe and effective results.

## Introduction

Acute aorto-iliac occlusion is an emergency critical disease with high risk of amputation and life-threatening complications (1–3). Previous studies reported on a substantial amount of mortality was as high as 75% (4). Acute aorto-iliac occlusion was caused by embolic migration or /and local thrombosis. Cardiogenic embolism was the main source of arterial emboli (5). Bilateral transfemoral embolectomy with Fogarty catheter and catheter-directed thrombolysis (CDT) are the traditional treatments for acute lower limb ischemia (ALI) (4, 6). However, patients with ALI usually present with chronic cardiopulmonary diseases and with significant risk of reoperation and bleeding complications (7). Several percutaneous mechanical thrombectomy (PMT) devices are available for treating ALI recently. For artery occlude below the groin area, it has been proved as an effective device (8). It's endovascular and minimal invasive, and able to rapidly remove thrombus with low risk of bleeding. However, the literatures were limited of PMT for treating aorto-iliac occlusion. The present study was performed to evaluate the safety and efficacy of PMT for ALI caused by aorto-iliac occlusion using the Rotarex device.

## Materials and Methods

### Subjects

**Figure 1:**
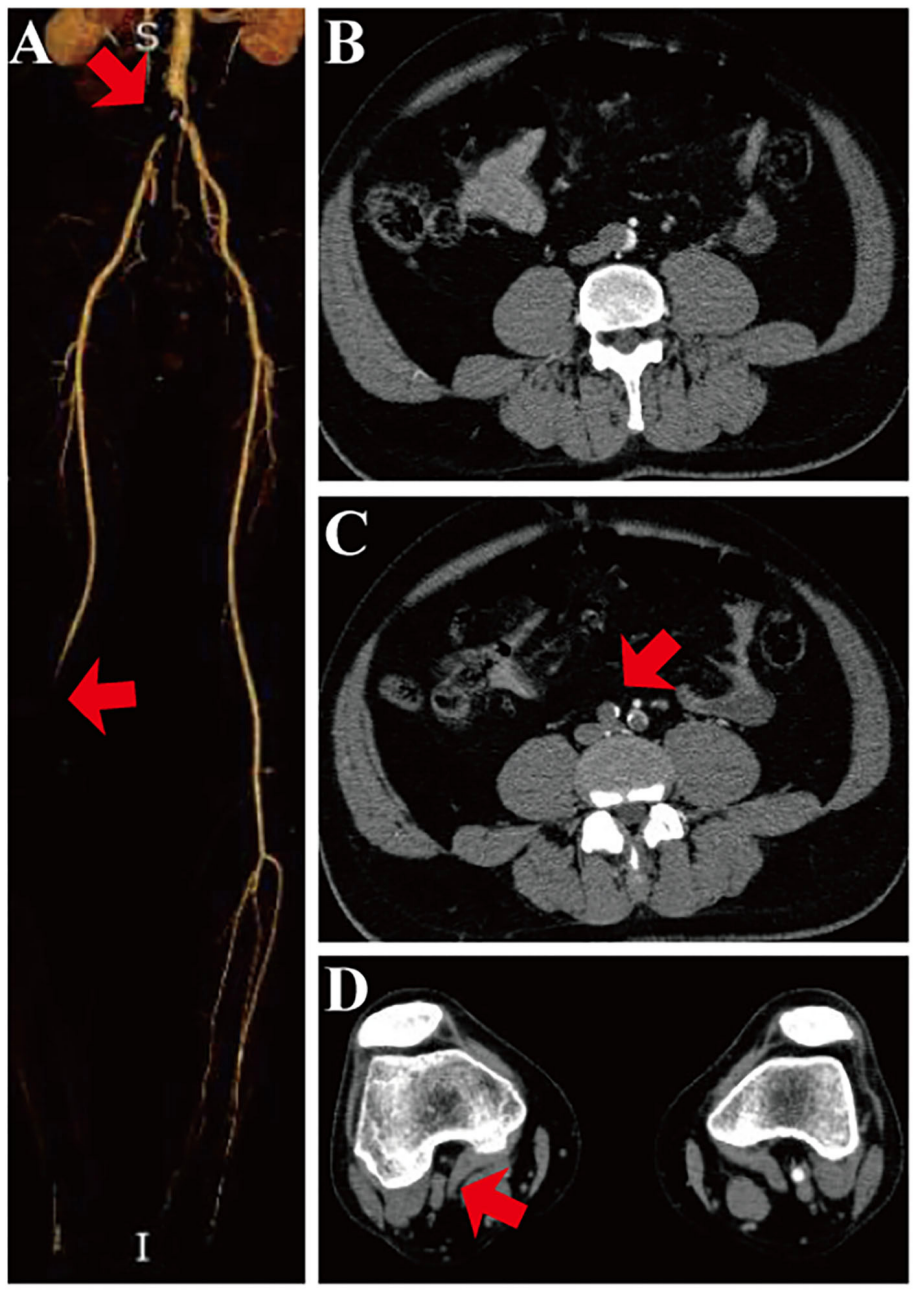
Preoperative CTA scan of the abdominal aorta and peripheral artery. **(A)** Three-dimensional CTA scan shows embolism (red arrow) in the inferior segment of the abdominal aorta, bilateral common iliac artery, and the right popliteal artery. **(B)** Cross-section of CTA shows embolism in the inferior segment of the abdominal aorta. **(C)** Cross-section of CTA shows embolism in the bilateral common iliac artery (red arrow). **(D)** Cross-section of CTA shows embolism in the right popliteal artery (red arrow).

A total of 342 patients were diagnosed as ALI between January 2010 and April 2020. Among them, 85 were caused by aorto-iliac occlusion with computed tomography angiography (CTA) revealing occlusion in the lower part of the abdominal aorta, and extended to bilateral iliac arteries (Figure 1). Thirty-eight patients treated with PMT (Rotarex system) were enrolled in present study. The ethics committee of local medical institution approved the study protocol and waived individual patient consent for the present retrospective analysis.

### Device and Procedure

All procedures were performed during emergency intervention after diagnosis. After local anesthesia, introducer sheath was inserted through the femoral artery or left brachial artery approach. Bilateral femoral artery approaches or crossover technique from one femoral artery approach was used according to the operator. Heparin sodium was administered at 80 IU/kg to achieve an activated clotting time of 250–300 s in all patients. A 0.35-inch stiff wire (Terumo Corp., Leuven, Belgium) supported by a 4F multipurpose catheter was used to pass through the occluded lesion. Arteriography was performed to confirm that the catheter tip was located in normal artery lumen.

**Figure 2:**
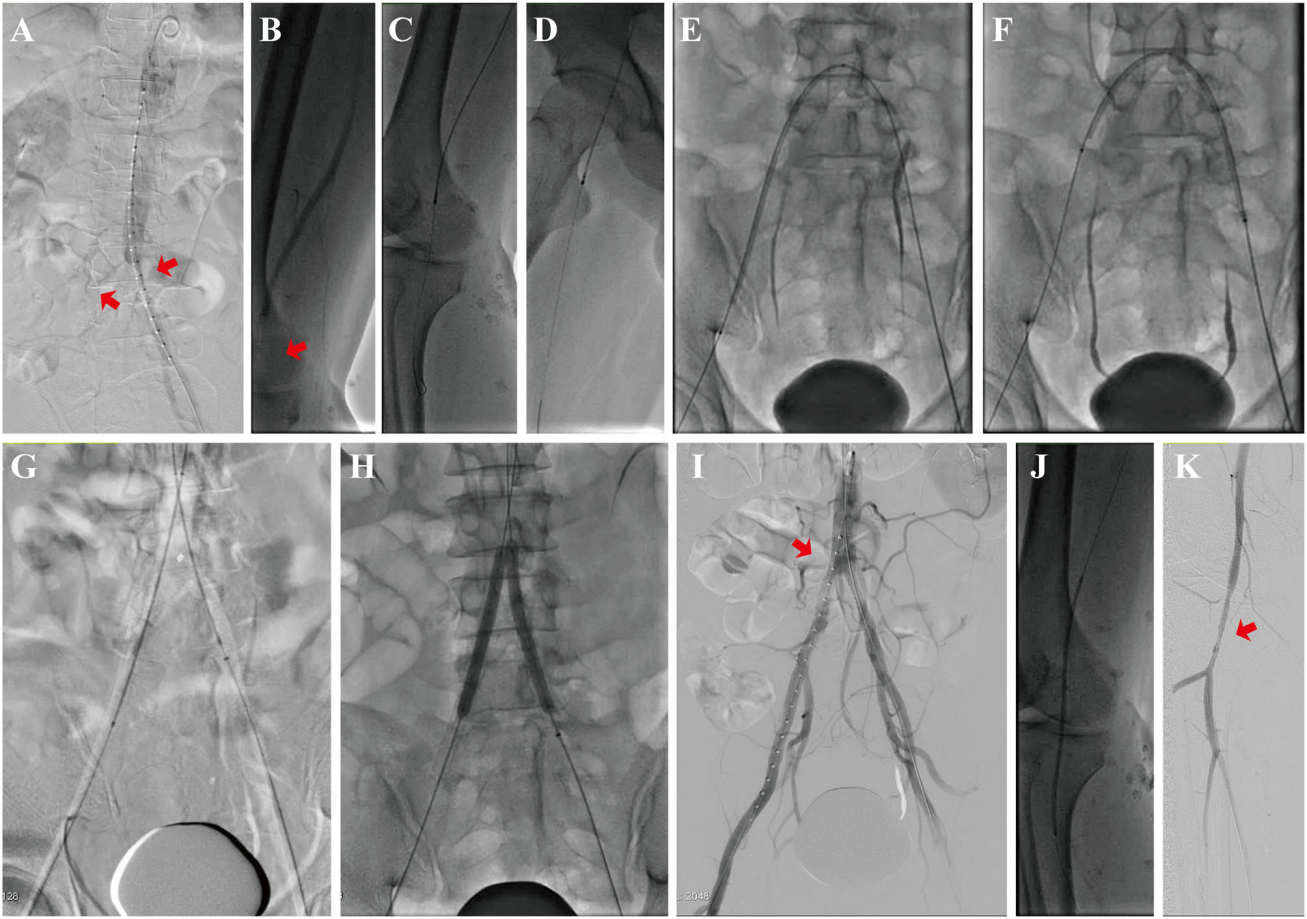
Mechanical thrombectomy process by using the Rotarex® catheter for AASE treatment. **(A)** Emergency DSA shows occlusion of the distal aorta (red arrow) and bilateral common iliac artery. **(B)** Angiography shows occlusion of the right popliteal artery (red arrow). **(C)** Mechanical thrombectomy of the right popliteal artery. **(D)** Mechanical thrombectomy of the bilateral common iliac artery. **(E)** Balloon dilatation of the right iliac artery. **(F)** Balloon dilatation of the left iliac artery. **(G)** Kiss stenting of the bilateral common iliac artery. **(H)** Balloon dilatation of the bilateral common iliac artery. **(I)** Angiography shows the patency of the bilateral common iliac arteries (red arrow). **(J)** Balloon dilatation of the right popliteal artery. **(K)** Angiography shows the patency of the right popliteal artery (red arrow).

The Rotarex system is a mechanical endovascular thrombectomy device that can rotate up to 40,000–60,000 rpm (9, 10). This condition creates a powerful vortex that can debulk all detachable occlusion materials in the artery. The catheter head has side slits, which subsequently enunciate the fragmented debris, while the inner helix simultaneously generates a strong suction force. Finally, this force helps transport the fragmented materials into an external collection bag. A 6-Fr or 8-Fr Rotarex catheter was used, depending on the vessel size. 8-Fr was commonly used for iliac vessels and the aorta. Slowly forward and backward motion was performed with 1–2 cm per second until the distal end of the occlusion was reached as previously reported (11). Crossover technique was applied in 16 (42.1%) procedures. Additional runs were conducted to aspirate all the thrombotic materials when necessary. Balloon angioplasty and/or stent implantation were used when the residual stenosis was over 30%. Covered stent was preferred to avoid bleeding after PMT and balloon angioplasty. Frequent flushing of the device outside the patient is mandatory to avoid any dysfunction such as sticking the catheter to the guidewire or breaking the helix. The catheter system can not only for iliac vessels but also fem-popliteal artery. If necessary, multi-level thrombectomy was performed. If thrombus dropped into distal arteries, adjunctive manual aspiration with a 6F guiding catheter was used to aspirate the thrombus (Figure 2).

### Anticoagulation and Antiplatelet Therapy

After the aorto-iliac occlusion diagnosis, anticoagulation treatment was immediately initiated. Low-molecular weight heparin (1 mg/kg) twice at an interval of 12 h. A lifelong anticoagulant therapy of rivaroxaban (15 mg rivaroxaban orally twice a day for 3 weeks, and then 20 mg rivaroxaban once a day) or warfarin (with international normalized ratio (INR) of 2–3) was administered for patients with atrial fibrillation and rhematic valvular disease when discharged according to the guideline (12). In addition, patients with balloon angioplasty and/or stent implantation were instructed to take clopidogrel (75 mg/d, 6 months) and lifelong aspirin (100 mg/d) therapy.

### Follow-Up

**Figure 3:**
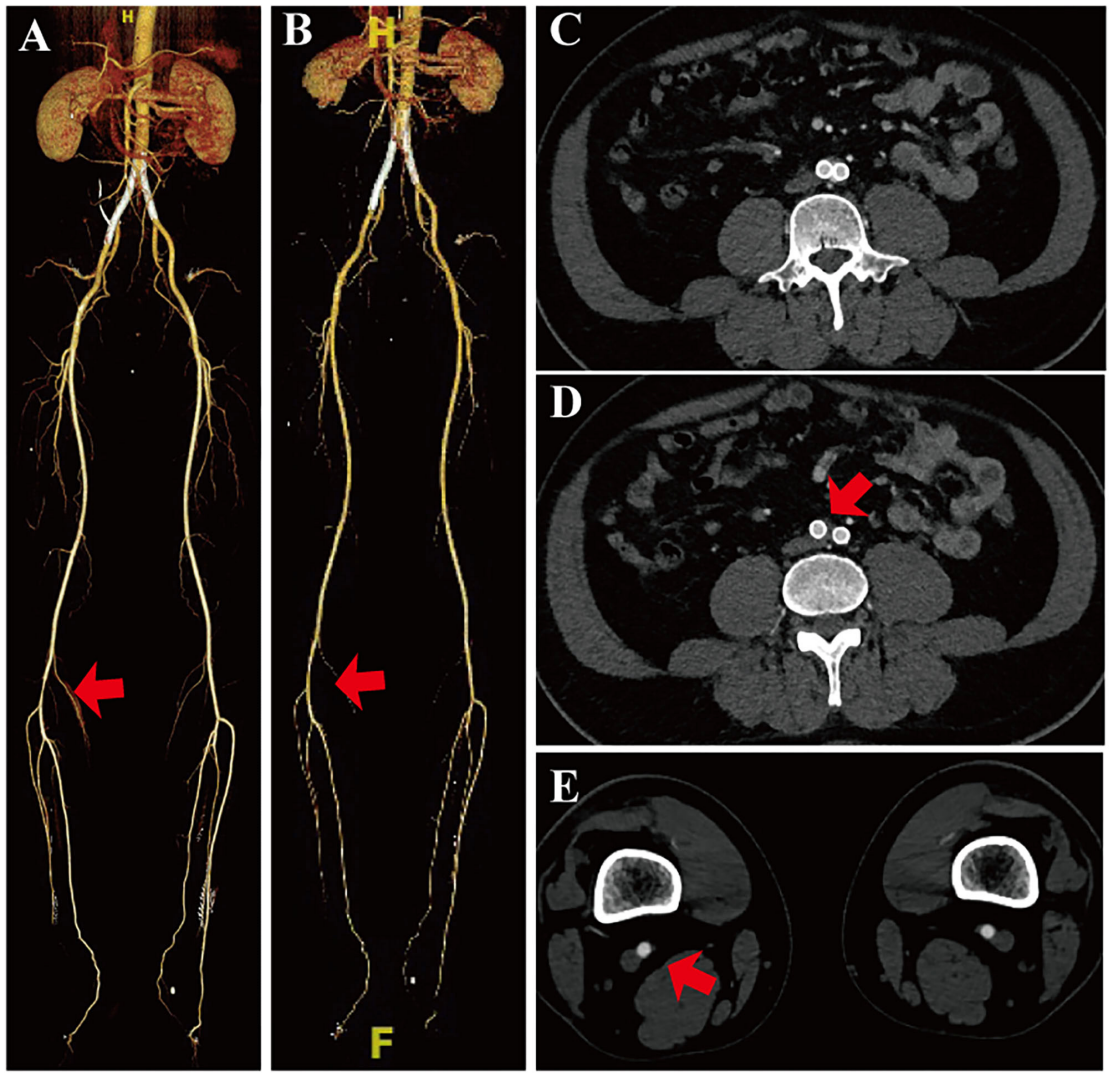
Follow-up CTA of the same patient. **(A)** CTA shows the patency of the bilateral common iliac artery and the right popliteal artery after 6 months (red arrow). **(B)** CTA shows the patency of the bilateral common iliac arteries and the right popliteal artery after 18 months (red arrow). **(C–E)** Cross-section of CTA of 18 months.

The patients were followed up at 30 days, 6, 12 and 18 months. A routine outpatient Doppler ultrasound examination and CTA were performed (Figure 3). The primary outcome was the limb salvage rate, which was defined as freedom from above-the-ankle amputation. The secondary outcomes included postoperative mortality, the target artery patency rate, and other postoperative complications.

### Definitions

ALI was defined as the onset of symptoms within 14 days. ALI was classified as grade I, IIa, IIb, and III following the Rutherford classification criteria (13). Amputation was inevitable for patients with grade III ischemia, therefore patients with grade III ischemia were not included in present study. Technical success was defined as complete aortic bifurcation thrombosis removal from the iliac arteries and a residual stenosis <30% at the end of the procedure. Renal insufficiency was defined as a glomerular filtration rate <60 ml/min. Major complications included death and amputation. Minor complications included reversible renal injury, osteofascial compartment syndrome, Rotarex-induced perforation, and distal embolism.

### Statistical Analysis

Continued data were expressed as mean values ± standard deviation. Categorical data were reported as proportions. Significant differences were considered at *P* < 0.05. Data were analyzed using the SPSS 20.0 software.

## Results

### Patient Demographics

Among the 38 ALI patients with aorto-iliac occlusion, 24 (63.2%) were male. The mean age was 66 years old (28–83). Comorbid medical conditions were presented in Table 1. Over 90% of patients were diagnosed with atrial fibrillation and rhematic valvular disease. Arterial embolism was the main cause of ALI in current study. Duration from onset to operation was 10.3 (3–96) h. There were 2 (5.3%) patients classified as the lower limb ischemic stage class I, 16 (42.0%) in the lower limb ischemic stage class IIa, 20 (52.6%) in the lower limb ischemic class IIb. All 38 patients were treated with PMT with additional catheter directed thrombolysis using urokinase (2/38, 5.3%), balloon angioplasty (8/38, 21.1%) and stent deployment (7/38, 18.4%). The mean time of the interventional procedure was 123 ± 31 min. The technical success rate was 78.9%. With the additional methods, the success rate was 100%. Seven patients (18.4%) underwent continuous renal replacement therapy (CRRT) for severe reperfusion injury, electrolyte disorders, and renal insufficiency during or post operation.

**Table 1:** Patients' demographics.

**Variables**	**Mean (range) or percentage (*N* = 38)**
Age (years)	66 (28–83)
Male	24 (63.2%)
Comorbidity	
Hypertension	21 (55.3%)
Diabetes mellitus	20 (52.6%)
Atrial fibrillation	32 (84.2%)
Rhematic valvular disease	3 (7.9%)
Coronary artery disease	17 (44.7%)
Smoking	20 (52.6%)
Renal insufficiency	7 (18.4%)
COPD	5 (13.1%)
Phase of limb ischemia	
Class I	2 (5.3%)
Class IIa	16 (42.0%)
Class IIb	20 (52.6%)

*COPD, Chronic obstructive pulmonary disease*.

The mean postoperative length of stay was 8 ± 4 days, the 30-day survival rate was 94.7%, and limb salvage was 94.4%. There were 8 complications. Two patients received major amputations (above the knee) and 2 patients died for renal insufficiency and heart failure. Other complications were Rotarex-induced perforation (*n* = 1), osteofascial compartment syndrome (*n* = 2), and blue toe syndrome due to distal embolism (*n* = 1). Perforations were treated successfully with covered stent deployment. Osteofascial compartment syndromes were treated with fasciotomies. Blue toe syndrome was treated with medication (Table 2).

**Table 2:** Operative details.

**Operative details**	**Mean (range) or percentage (*N* = 38)**
Technical success	38 (100%)
Balloon angioplasty	8 (21.1%)
Stent deployment	7 (18.4%)
Operative time, min	123 ± 31
Dialysis treatment	7 (18.4%)
Complications in 30 days after operation	
Major amputation	2 (5.3%)
Death	2 (5.3%)
Perforation	1 (2.6%)
Osteofascial compartment syndrome	2 (5.3%)
Blue toe syndrome	1 (2.6%)
Length of stay, d	8 (4–28)

The mean follow-up time was 14.0 months (range 8–22 months). Two deaths resulted in a survival rate of 92.1%. One patient died of pulmonary infection at 8-month, one patient died of myocardial infarction at 21-month. There was no major amputation and target artery occlusion occurred during the follow-up period.

## Discussion

ALI is a dramatic event with high risk of amputation, morbidity and mortality. And more proximal location of clot causes a severe ischemia due to the lower possibility of compensation by the collateral circulation. Acute aorto-iliac occlusion could lead to limb ischemia, tissue necrosis, even amputation and /or death due to severe reperfusion injury and rapid multiple organ failure (2, 3, 14). A rapid diagnosis and management are important (15). The present study showed PMT with additional methods was safe and effective with satisfactory limb salvage rate for acute aorto-iliac occlusion.

The traditional intervention of ALI was thromboembolectomy with Forgarty catheter. It is an efficient treatment for acute arterial emboli of lower limbs. However, the early clinical outcome still remains unsatisfactory in a number of cases (16), especially in diabetic patients. CDT has been used as an alternative to surgical embolectomy, especially when patients present with less severe ischemia (Class I and IIa). However, it has a significant risk of bleeding complication. The risk of bleeding was increased up to 19% when heparin was used simultaneously according to literature (17). PMT devices have been applied to shorten time consumption for dissolution of thrombus and to reduce the amount of thrombolytic agent used (18). The Rotarex catheter is a commonly used device and has been proved to be safe and effective in the treatment of peripheral artery diseases with 95% success rate (19). It could simply and directly remove the blood clots and restore the blood flow quickly without delaying the treatment compared to the conventional operation procedure (8, 20). It is feasibility in patients with high risk of bleeding when thrombolysis in contraindicated (hepatic failure, recent surgery, trauma, or a neurovascular accident) (21). The technical success and limb salvage rate were satisfactory in present study. Additional methods, such as balloon angioplasty and stent implantation, were necessary when PMT couldn't achieve enough lumen gain.

The other PMT device used in our center was the Angiojet catheter. The AngioJet is a rheolytic thrombectomy device made of a double-lumen over-the-wire catheter that uses the Bernoulli's principle for thrombus aspiration. However, the rapid stream of fluid and hydrodynamic forces used by thrombectomy devices may cause significant amount of red blood cell hemolysis resulting in hemoglobinemia and hemoglobinuria (22). This may occur when repeated passes of the device are required, resulting in severe consequences, mainly in patients with renal insufficiency (23). In addition, for cases caused by arterial emboli, thrombolysis therapy with the Angiojet system is not always wholly efficient in dissolving the thrombus (24).

Rapidly recanalization is important in terms of limb salvage. Meanwhile recanalization following limb ischemia may result in reperfusion syndrome, which causes very high comorbidity and mortality (25). Ischemia-reperfusion injury is still a challenge for the treatment of ALI. All patients were treated with bicarbonate and mannitol to prevent acute renal failure, and 7 received CRRT. Initiation of CRRT at early stage may not only reduce the damage of renal function but also prevent other organ disfunction, increase the survival rate (26).

One device-related complication of Rotarex catheter is the distal embolization of the residual thrombotic debris in the peripheral arteries (27, 28). Some studies have shown that embolism-protective filters may reduce the incidence of distal arterial embolism. But routinely used of filter is not necessary (29). The incidence of distal embolism was 5 cases. This condition can be successfully treated by manual aspiration with a guiding catheter. Another identified complication related to the Rotarex catheter is vessel dissection or perforation, especially in small arteries (≤ 3 mm in diameter) and heavily calcified plaque (30, 31). Such complications can be remedied by prolonged balloon dilatation or covered stent implantation in most cases. In terms of aortic bifurcation and iliac artery, the incidence was rare but when perforation occurred, covered stent should be implanted immediately.

This study has several limitations. First, this is a retrospective study with a small sample size and short follow-up period. Thus, more clinical data is required. Second, the risk factors that affect the limb salvage and survive were not evaluated because of the small sample size of the study. Therefore, a larger sample size is needed to assess these risk factors.

## Conclusion

PMT with Rotarex catheter can be an alternative for acute aorto-iliac occlusion with satisfactory limb salvage and acceptable complications.

## Data Availability Statement

The original contributions presented in the study are included in the article/supplementary material, further inquiries can be directed to the corresponding authors.

## Author Contributions

XL, WL, and JQ: conception and design. XY, MY, RW, KY, and YC: analysis and interpretation. XY and XL: writing the article. XY, XL, YC, WL, and JQ: critical revision of the article. XY and RW: statistical analysis. All authors contributed to the article and approved the submitted version.

## Funding

This study was financially supported by the National Natural Science Foundation of China (81971758, 81971712) and the Natural Science Foundation of Shanghai Science and Technology Committee (Grant No. 20ZR1431600).

## Conflict of Interest

The authors declare that the research was conducted in the absence of any commercial or financial relationships that could be construed as a potential conflict of interest.

## Publisher's Note

All claims expressed in this article are solely those of the authors and do not necessarily represent those of their affiliated organizations, or those of the publisher, the editors and the reviewers. Any product that may be evaluated in this article, or claim that may be made by its manufacturer, is not guaranteed or endorsed by the publisher.
